# Production of Open-Cell Metal Foams by Recycling of Aluminum Alloy Chips

**DOI:** 10.3390/ma16113930

**Published:** 2023-05-24

**Authors:** Sonja Jozić, Branimir Lela, Jure Krolo, Suzana Jakovljević

**Affiliations:** 1 Faculty of Electrical Engineering, Mechanical Engineering and Naval Architecture, University of Split, Rudjera Boškovića 31, 21000 Split, Croatia; 2 Faculty of Mechanical Engineering and Naval Architecture, University of Zagreb, Ivana Lučića 5, 10000 Zagreb, Croatia

**Keywords:** open-cell foam, recycling, machining chips, solid state, space holder agent, aluminum

## Abstract

In this paper, an innovative sustainable method of producing metal foams was presented. The base material was aluminum alloy waste in the form of chips obtained by machining process. The leachable agent, used to create pores in the metal foams, was natrium chloride, which was later removed by leaching, resulting in metal foams with open cells. Open-cell metal foams were produced with three different input parameters: volume percentage of natrium chloride, compaction temperature, and force. The obtained samples were subjected to compression tests during which displacements and compression forces were measured to obtain the necessary data for further analysis. To determine the influence of the input factors on the selected response values such as relative density, stress and energy absorption at 50% deformation, an analysis of variance was performed. As expected, the volume percentage of natrium chloride was shown to be the most influential input factor because it has a direct impact on the obtained metal foam porosity and thus on the density. The optimal values of the input parameters with which the metal foams will have the “most desirable” performances are a 61.44% volume percentage of natrium chloride, a compaction temperature of 300 °C and a compaction force of 495 kN.

## 1. Introduction

Metal foams represent a relatively new type and form of materials that was developed based on imitating the structure and properties of natural cellular materials such as wood, bone, coral, and honeycomb. Their most important properties are based on the type of cell wall material, the shape of cells and the production process by which they were obtained [1]. Metal foams are most often required to retain all useful properties of the base metal. The coefficient of thermal expansion of the foam is also close to the coefficient of the base metal, whereas their thermal conductivity decreases due to the porous internal structure [2]. Over the years, a number of patents have been filed in the field of metal foam production. Despite all of the above, so far, a relatively small number of commercial manufacturers in the world deal with closed- or open-cell metal foams [3].

According to their structure, metal foams are divided into two groups: closed-cell foams, where the cells are separated from each other by thin walls, and open-cell foams, where the cells are made of supports, not walls, so the internal parts of the cells are interconnected. In addition to cell types, metal foams are characterized by size, shape and degree of cell anisotropy, material type and relative density (ratio of foam density to the density of the base material).

The application of aluminum foams is diverse, and the most common areas of use are: structural elements in lightweight structures, energy-absorbing elements, vibration and sound-damping elements, filters, design elements, reinforcement components and heat exchanger elements [4]. Furthermore, since it is possible to produce foams of very high density, and thus small dimensions of intercellular spaces, the structure of open cells is extremely suitable for making filters for liquids and gases [5]. Guarino et al. [6] developed an innovative heat exchanger manufactured by using open-cell Al-based foams. Ubertalli and Ferraris [7] and Bauer et al. [8] reported on Al metal as a raw material for the production of permanent cores in different casting processes. Intensive development and efforts to adopt production are justified by a possible very wide field of application-for parts of road and rail vehicles, aircraft, ships, construction, furniture, decorative items, medical aids, heat exchangers, sound and vibration dampers, etc. [9]. For wider application, the limitation is still the high cost of metal foams. Finding new, more efficient and better controllable foam production processes is necessary in order to achieve even better properties. This is also important so that the production process, based on predetermined parameters, achieves precisely defined properties [10].

Metal foams with open cells are most often produced using a solid space holder and thermal treatment such as sintering and solidification of molten metal. The empty space in the final structure is formed after burning out the space holder or the leaching of the soluble substance [11]. Among all metals, Al-alloy foams are mostly in the focus of research by scientists and industry, due to their very favorable properties such as specific mechanical and physical properties [12]. Ozer et al. [13] described, in their paper, a method of producing open-cell aluminum foams, for which they used as the space holder furan bonded sand and gypsum bonded ceramics, whereas the metal matrix was aluminum alloy A413. Finkelstein et al. [14] showed the production methodology of highly porous replicated aluminum foam using a double-granular space holder. Sutygina et al. [15] have presented the manufacturing of open-cell copper foam using the sponge replication technique. As a basic raw material, they used copper powder and an open cell was formed by dipping polyurethane (PU) foam into the slurry consisting of copper powder, binder and water. The last stage in the foam production process is drying and burning out of the binder and PU foam. To produce open-cell structures, Cadenaa et al. [16] applied Al powder with 99.5% purity and diameters between 75 µm and 200 µm and the space holder particles were spherical carbamide CH_4_N_2_O with a diameter from 1 mm to 2 mm at a sintering temperature of 620 °C. Daoud et al. [17] presented the fabrication process of quality composite foams by using recycled aluminum alloy 7075 beverage cans and silica waste particles as reinforcements and CaCO_3_ as a foaming agent. Open-cell aluminum foams can be produced by infiltrating liquid aluminum through the pore spaces between loose compacts of natrium chloride beads [18].

Today’s requirements for sustainable production are visible in all branches of production, including the production of metal foams. In his publication from 2013, Banhart [19] presented alternative processes for precursor of foam production on the basis of Al–Mg–Si machining chips. Since the most common base material for fabricating metal foam, i.e., metal powder, is expensive, it is recommended to use recycled metal instead, e.g., foil, chips or cans. Clearly, these are cheap sources of raw material so their composition is not constant and also difficult to control. The morphology of such material is different from the morphology of atomized powders. Aluminum machining chips are usually problematic for conventionally recycle due to their small dimensions, high surface to volume ratio, and increased oxidation, but in metal foam production, this major drawback could be used in order to omit metal comminuting step into powder.

According to the authors, very few papers have been published dealing with the production of metal foams from aluminum scrap, and the most common method is the re-melting of aluminum chips with the addition of cell-forming agents. A blowing agent was more commonly used for closed-cell foam production but space holding (leachable) agents were also used for open-cell metal foam production in some cases.

Haesche et al. [20] presented a foam production process based on thixocasting of compacted metal chips. As the foaming agents, they used CaCO_3_ (lime) and CaMg (CO_3_)_2_ (dolomite) and examined the pore structure and density of the samples at different foaming temperatures. Kumar et al. [21] reported on aluminum foam produced from re-melted metal chips with the addition of TiH_2_ and alloying with Mg while keeping the mixture in a liquid state to allow reaction and oxide fragmentation. Kanetake et al. [22] investigated the influence of different shapes, sizes and chemical composition of aluminum chips on the foaming behavior. A. Osman et al. [23] presented a very detailed and exhaustive analysis of the properties of aluminum foam produced from aluminum scrap using CaCO_3_ as the foaming agent. The density of aluminum foam was in the range from 0.40 g/cm^3^ to 0.60 g/cm^3^ and the relative density from 0.14 to 0.25. Tsuda et al. [24] concluded that the production process of precursor, the content of TiH_2_ and the addition of the ceramic particles have a decisive influence on foam porosity and shape of the cells. Rivera et al. [25] reported on aluminum alloy foams manufactured from the recycling of cans. Foams were prepared by the melting process of the A242 aluminum alloy and adding TiH_2_ which served as a blowing agent. Jamshidi-Alashti et al. [26] suggested a method based on semisolid metal processing. This is a semisolid melt-squeezing production procedure. Preheated NaCl particles were added to the molten A356 alloy. This mixture was solidified by stirring to the desired solid fraction of primary particles. The mixture was then compressed and finally an open-cell foam was obtained by washing the final Al-Si-NaCl composites in water and removing NaCl that served as space holding agent. Moloodi and Raiszadeh [27] presented the research results of the sintering-dissolution process for the open-cell aluminum foam production process. They used five different sizes of aluminum chips obtained in turning as the base material and NaCl as space holding agent. They concluded that chip weight fraction of 60%, chip size of about 0.5 mm and a sintering temperature of 600 °C to be the optimal parameters for a successful sintering dissolution process for the fabrication of aluminum foams from AA336 turning scrap.

The main aim of this research was to investigate metal foam production from aluminum chips and space holding (leachable) agents in the form of the NaCl particles. The mixture of aluminum chips and NaCl particles was compacted at room and different elevated temperatures. After the compaction step, the obtained precursors were placed in ultrasonic bath and after that in boiling water with the intention of removing NaCl particles and to obtain open-cell metal foams. Therefore, the aluminum chips were recycled without the re-melting process. The possibility of mutually obtaining low-cost and open-cell aluminum foams in solid-state chips recycling was evaluated.

Furthermore, with the aim to investigate the influence of production parameters, design of experiments approach was utilized. The influence of the process parameters (volume percentage of space holder, compaction force and temperature) on utility properties (relative density, stress, and energy absorption) was determined and described by statistical analysis using regression and variance analysis. Optimal production input parameters were proposed according to the statistically obtained mathematical models. To obtain insight into the microstructure of the metal foam sample, a scanning microscopic analysis of the metal foam that was finally produced with optimized parameters was performed.

## 2. Materials and Methods

**Figure 1:**
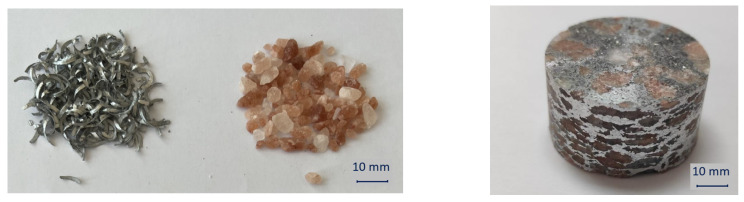
Aluminum alloy EN AW 2011 chips and Himalayan salt used in experiments and compacted precursor.

In this research, the metal foam base material was aluminum alloy EN AW 2011 in the form of chips whose chemical composition is given in Table 1. Machining chips were obtained by a milling process in which cold compressed air was used as a cooling medium, so chips were not contaminated with cooling and lubrication fluid. The chips were obtained out of EN AW 2011 block with dimensions of 100 × 100 × 150 mm. All chips had the same volume because they were obtained with the same milling parameters, Figure 1.

Natrium chloride (NaCl), in the form of the Himalayan salt with an average particle size of 5.2 mm, was used as a space holder agent. The machining chips and the space holder agent were mixed with varying volumes of the NaCl in range from 50% to 70%. It was expected that the volume percentage of the space holder agent would correspond to the porosity in the obtained metal foam if the leaching was to be successful.

To obtain compacted briquette precursors, the mixture (chips, NaCl and a small amount of distilled water) was placed in a steel mold with a diameter of 38 mm and compacted with a force of 300 kN in a hydraulic press at room temperature. The HBM C6A force sensor was used for measuring the value of the compacting force. The next step was additional compaction of the precursors in order to investigate the influence of compaction forces in range from 300 kN to 500 kN at different temperatures in range from 20 °C to 300 °C. Additional compaction was performed in a steel mold with a diameter of 40 mm and therefore additional plastic deformation was introduced into samples to achieve better connection between machining chips. As well known from previous research dealing with solid-state aluminum recycling, elevated temperature, forces and plastic deformation should serve for better chips connection and therefore better-quality metal foams [28,29].

The process of forming solid precursors was followed by its cleaning from space holder agents in order to form porosities in the base material and thus obtain a metal foam with the desired porosity. This is the reason why space holder agents are sometimes also called leachable agents. The space holder agent cleaning process was performed in an ultrasonic bath at 60 °C temperature and afterwards in boiling water for 30 min.

**Figure 2:**
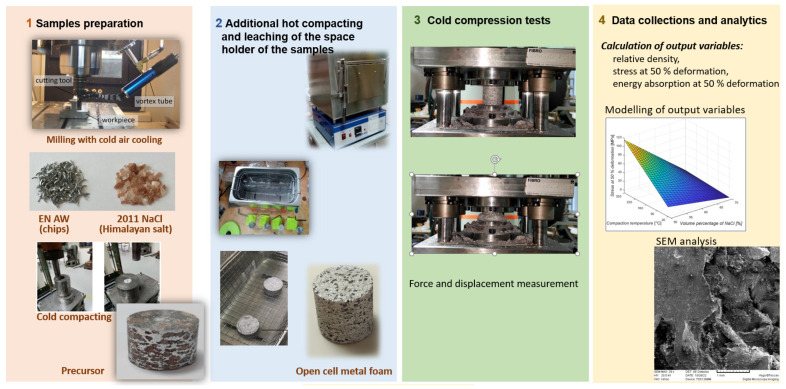
Presentation of activities in the experimental part of the work.

After chips-based metal foams were produced, all samples were subjected to a cold compression test, during which displacement and force were measured. The measured force and displacement values were later used to evaluate the performance of the individual samples. Figure 2 shows the diagram of the activities in the experimental part of the work.

The goal of hot compaction at different temperatures and different forces is to gain insight into whether these two parameters affect the output parameters, i.e., the properties of metal foams.

Though literature research and numerous preliminary tests, the influential input variables as well as their range of values were determined. The influential input variables were the volume percentage of the space holder (NaCl) which will have a direct influence on metal foam porosity, compaction temperature and compaction force, the minimum and maximum values of which are shown in Table 2. The most important goal of the experimental part, and at the same time of the entire work, is to determine the influence of the input factors on the properties of the produced aluminum foams.

As with the preliminary test, the samples were exposed to the stages of cold compaction (briquetting), hot compaction, leaching, and at the end cold compression test, during which forces, displacements and dimensions of the samples were measured, which was the basis for the calculation of output values. Prior cold compression test sample weight and outer dimension were measured to calculate relative density. The observed output values of the process are explained below.

Among the main characteristics of metal foams is their extremely low volume compared to other materials, so density and relative density are taken as relevant quantities that describe metal foams well. The following expression is used to calculate the relative density *ρ_rel._* of metal foams:

*ρ_rel._* = *ρ_mf_*/*ρ_Al_* (1)

where *ρ_mf_* is density of metal foam and *ρ_Al_* is density of aluminum alloy, (2.7 g/cm^3^). As expected, the most important input factor in this output variable is the amount of porosity in the structure, and the possible influence of other two input factors will be considered later in the statistical analysis of experimental data.

**Figure 3:**
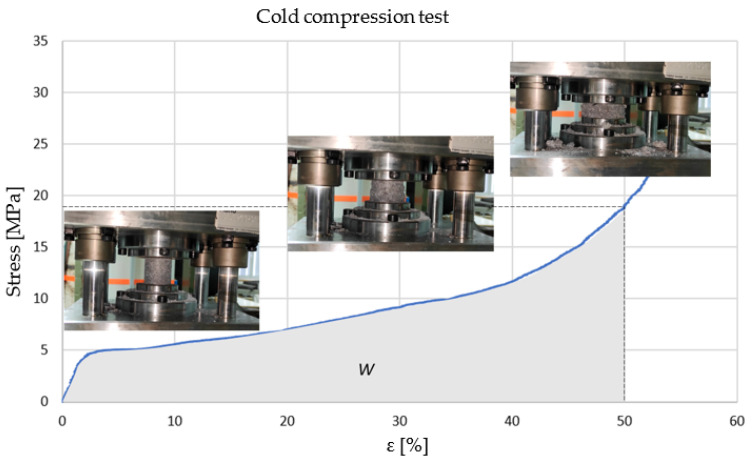
Cold compression tests and engineering stress–strain curve for Sample nr.1.

The measured force–displacement data were used to determine the engineering stress–strain curve. Figure 3 shows the stress–strain diagram, which consists of an elastic part, a stress deformation plateau, which has a smaller slope than the elastic part, and a part where foam begins to collapse. The stress value for deformations of 50% is marked on the diagram as well, and the precise values are calculated. Due to the different behavior of differently obtained open-cell metal foams, a constant engineering strain value was selected at the ending part of the plateau stress at 50%. The reason for choosing a constant engineering stress is to compare obtained stresses at that constant strain for each foam and to compare them in statistical analysis. The diagram shows that the stress for most foams during compression has a sudden increase after a deformation of 50%, which indicates the densification of the foam during compression and the consequent rupture of internal bonds and foams cells collapse. Therefore, one could fairly state that examination of the stress after engineering strain higher than 50% has no practical use. The shape of the metal foams cells is approximately defined by the mathematical model of the tetrakaidecahedron polyhedron and this makes it insensitive to the direction of stress. This cells shape allows the metal foams to absorb impact energy from any direction. Foams with equal size and periodic distribution of cells can be considered isotropic; however, during processing and production, irregularities always occur in the arrangement of the cells, and the cells are oriented in a certain direction, their volume increases and this results in unequal cell sizes.

The two most important factors for energy absorption are the relative density of the metal foam and its porosity. Foams with smaller pores contain more material and a larger surface area, which is why when subjected to stress or force, they absorb larger amounts of energy than foams with larger pores. Foams with a higher pore density are more resistant to stress. The relative density determines the thickness of the joint between the two nodes of the cell, because the thicker it is, the cell can absorb more energy and the metal foam is stronger.

Energy absorption at 50% deformation can be determined using the stress–strain diagram, since the area under the curve in the diagram is equal to the amount of absorbed energy, colored grey in Figure 3. The deformation of 50% will be taken as the densification limit for measurement. Calculating the area under the curve the absorption energy per unit volume for a sample can be evaluated by means of the equation:

(2) $$W=\int_{0}^{\varepsilon_{50\%}}\sigma \left(\varepsilon \right)d\varepsilon$$

where *σ* i *ε* represent compression stress and strain, respectively. Ideal energy absorbers have a long, flat stress–strain or load–deflection curve, so the absorber collapses plastically at a constant stress called the plateau stress, *σ_pl_*.

In this study, the experiments were conducted according to the D-optimal experimental plan based on the response surface method. The D-optimal method is carried out with a smaller number of experiments compared to other factorial experimental plans. In addition to numerical factors, categorical factors can be included in the experimental plan. When creating experimental points, it remains within the upper and lower values of the input factors, which is very important for the implementation of experiments and the physical limitations of the equipment and the process itself.

There are various criteria for the design of an optimal plan, among which the D-optimality criterion is the most popular, since it aims to maximize the determinant of the information matrix, (X′X), while providing the best simultaneous estimation of the model parameters. Here, X is a design matrix with n rows representing the experiments in the design and p columns representing the model coefficients. Additional information on the D-optimal method can be found in the book of Mead et al. [30].

The experimental design has three numerical factors (volume percentage of space holder, compaction temperature and compaction force) whose combinations and values (within the given limits, Table 2) were determined using a statistical package, Design Expert 8. The experimental design has a total of 20 experiments, of which 5 experiments are replicates to estimate pure error and 5 experiments are required to estimate lack of fit.

All experimental runs together with the experimentally determined and calculated output variables (relative density, stress value and energy absorption for deformations of 50%) are shown in Table 3. The volume percentage of NaCl, compaction temperature and compaction force are now labeled as A, B and C, respectively.

The calculated values of the output variables were entered into the Design Expert 8 program, where the influence of the input factors on the mentioned output variables was analyzed. Response surface methodology was used to obtain the mathematical dependence between responses and input parameters. The suitability of the model within the used program is based on the F-test and the value of the coefficients of determination R^2^. An analysis of variance (ANOVA) was performed for each output variable, which determines the level of significance of the included members that should make up the mathematical model. In the implementation of ANOVA, it is necessary to consider the Prob > F values determined for each member currently involved. If the Prob > F values for the included members are less than 0.05, such members are significant and should be included in the model, otherwise, if the Prob > F is greater than 0.05, the members are not significant and can be excluded from the analysis.

For a high-quality and complete analysis of the model and the selection of appropriate members that will make up the model, it is necessary to take into account other quantities that describe the mutual relations of the variables. The coefficient of determination R^2^ (R-squared) is an estimate of the total variance, that is, a measure of deviation from the arithmetic mean. In addition to R^2^, the adjusted R^2^ (adjusted R-squared) and the predicted R^2^ (predicted R-squared) also differ, where the adjusted R^2^ is adjusted to the number of model members, while the predicted R^2^ is the size of the variations in the new data obtained by the model. As for the basic one R^2^, the closer the values of the additional coefficients of determination are to the one, the better the model is explained [30].

It should also be noted that the values of deviation from the model (lack of fit) are the variations in the measured quantity in relation to the average values of the central points, while the pure error is the error of repeating the state of the experiment for the purpose of estimating the variance.

## 3. Results and Discussion

### 3.1. Relative Density

According to the analysis of variance for the relative density of metal foams, shown in Table 4, it is evident that the volume percentage of the space holder and the compaction temperature, whose Prob > F values are less than 0.05, are significant input factors for the model, whereas the compaction force is not significant for the model. Input factors, regardless of their Prob > F values, cannot be excluded and will always be an integral part of the model even though they may not be significant to the model. Analysis of variance (ANOVA) showed that the density of metal foams was best described by a two-factor interaction (2FI) model. As can be seen from Table 4, factors A, B and AB are significant for the model.

**Figure 4:**
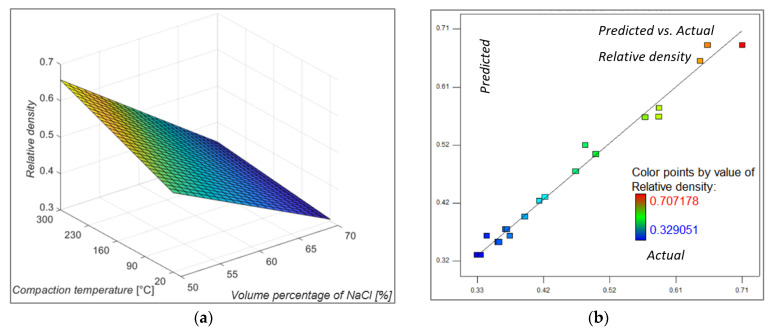
Model graph showing: (**a**) the influence of the volume percentage of NaCl and compacting temperature on relative density, compacting force at middle value; (**b**) predicted versus actual relative density values.

The values of the coefficient of determination R^2^, the adjusted R^2^ and the predicted R^2^ coefficient are 0.9861, 0.9797 and 0.9646, respectively. The coefficient values are relatively close to one, which indicates a relatively well-fitted model and less dispersion of the data. Figure 4a shows a change in the relative density of metal foams when the volume percentage of space holders and compaction temperature are changing. It can be seen that the largest influence on the relative density value is expected to be the volume percentage of space holders in the samples. The compaction temperature has a certain influence at lower values of the volume percentage of space holders but it loses its influence as the volume percentage of space holders increases. It is important to mention that a parameter that has a great influence on metal foam porosity in this research was the space holder agent cleaning step. If a small volume percentage of the NaCl particles was used there was a fair chance that some of the obtained pores will not be intersected. This will prevent the cleaning of those cells from NaCl and therefore those samples will most certainly have an increased relative density. Furthermore, those samples are expected to have increased stress values. That will also be emphasized at elevated temperatures because the flow stress of the base material is significantly decreased and as such there is a possibility that during the compaction step and deformation of the aluminum chips, aluminum will flow around the space holder agent and entrap some agent particles without possibility to efficiently clean them. That is an explanation of the influence of the elevated temperature on the relative density and why it loses its influence as the volume percentage of space holders increases.

Figure 4b shows the predicted versus actual relative density values, and it is obvious that these values are approaching each other. The mathematical model that can predict the relative density of metal foams is given by Equation (3).

(3) $$\rho_{rel}=1.02543-0.010335\cdot A+1.3644\cdot 10^{-3}\cdot B-1.9956\cdot 10^{-3}\cdot C-1.73905\cdot 10^{-5}\cdot A\cdot B$$

### 3.2. Stress at 50% Deformation

According to the variance analysis for stress at 50% deformation shown in Table 5, it can be seen that for the model, the significant input factors are the volume percentage of space holders and compaction temperature whose values Prob > F are less than 0.05, while compaction force is not significant. Analysis of variances (ANOVA) showed that stress at 50% deformation was most suitably described with a two-factor interaction (2FI) model. As can be seen from Table 5, factors A, B and AB are significant factors for the model.

The values of the coefficient of determination R^2^, the adjusted R^2^ and the predicted R^2^ coefficient are, respectively, 0.8826, 0.8284 and 0.6461. The coefficient values are relatively close to one, which indicates a well-fitted model and less dispersion of the data.

**Figure 5:**
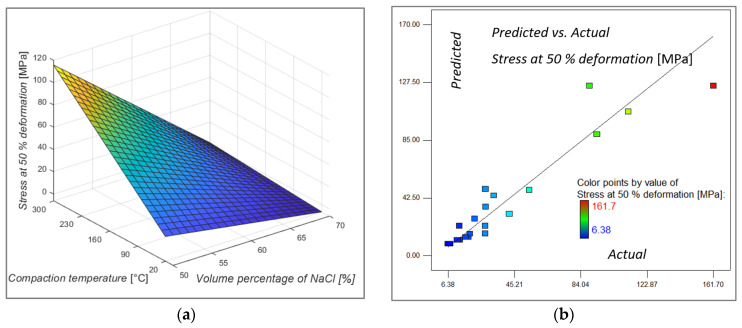
Model graph showing (**a**) the influence of the volume percentage of NaCl and compacting temperature on stress at 50% deformation, the compacting force at middle value (**b**) predicted versus actual value of stress at 50% deformation.

The 3D model of the response surface for stress at 50% deformation and the influence of input factors on it is shown in Figure 5a. The volume percentage of NaCl in the metal foams has a dominant influence on the stress values during this deformation. Samples with 50% NaCl in the structure have maximum stress values that decrease with increasing porosity (volume percentage of NaCl) in the structure. This model shows the interdependence of the factors when the compaction force has a value of 380 kN, but this input factor did not prove to be influential so that changing its value has a minimal effect on the model. Compaction temperature is another significant factor in addition to porosity. From the 3D representation of the surface, it can be seen that for samples with a porosity of 50%, the compression temperature significantly increases the stress value at a deformation of 50%. For samples with a higher proportion of porosity, the influence of compaction temperature loses its significance. Part of the explanation for this has already been mentioned in the section on relative density. As discusses for the lowest volume percentage of the NaCl and elevated compaction temperature there is a high probability that some of the NaCl particles will be entrapped between bonded aluminum chips. That is emphasized at elevated temperatures because aluminum chips can undergo more easily under plastic deformation and aluminum material will flow under high pressure more easily around the space holder agent and entrap some agent particles without possibility to efficiently clean them. Those entrapped particles are brittle and incompressible material surrounded with aluminum and during compression test for same displacement after collapse of the existing cells the stress will increase significantly. This is why those samples with a 50% volume percentage of NaCl particles which are compacted at elevated temperature have a drastic increase in stress values and relative density. This is also why it is always important to investigate influence of multiple input parameters and their mutual interaction on the novel production process. This is also why design of experiments methodology was used in this research. Predicted and actual values, shown in Figure 5b, are approaching each other.

The regression model for stress at 50% deformation, after omitting insignificant factors, is given by Equation (4).

*Σ at* 50% *deformation* = 48.33826 − 0.79103 · *A* + 1.17658 · *B* + 0.40185 · *C* − 0.016396 · *A · B* (4)

### 3.3. Energy Absorption at 50% Deformation

Analysis of the variance for energy absorption at 50% deformation determined that the input factors for the model are the influential volume percentage of NaCl and compaction temperature, the product of porosity and compaction temperature and the squared value of volume percentage of NaCl. In Table 6, analysis of variance and influential factors that meet the condition that Prob > F is less than 0.05 are presented. Analysis of variances (ANOVA) showed that the energy absorption at 50% deformation was most suitably described with a quadratic model. As can be seen from Table 6, factors A, B, AB and A^2^ are significant for the model.

**Figure 6:**
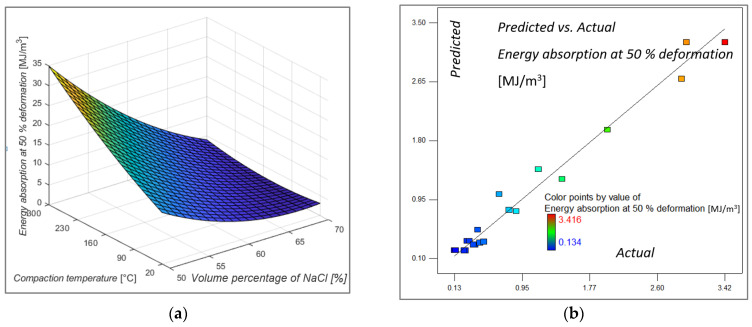
Model graph showing (**a**) the influence of the volume percentage of NaCl and compacting temperature on energy absorption at 50% deformation, the compacting force at middle value (**b**) predicted versus actual value of Energy absorption at 50% deformation.

As for other indicators, the values of the coefficient of determination R^2^ and the adjusted R^2^ and the predicted R^2^ coefficient are, respectively, 0.9769, 0.9561 and 0.8801, which indicates a very well-fitted model. Figure 6a shows the 3D model of the response surface for energy absorption at a deformation of 50%, the values of which are dominantly influenced by the proportion of porosity in the structure. The maximum values of absorbed energy are achieved for samples with 50% porosity in the structure, while the lowest values are achieved for samples with higher porosity, which correlates with the research of Fischer, [28]. Regarding the influence of compaction temperature on the response, it has a significant influence on the values of absorbed energy for samples with 50% to 60% porosity in the structure, and for samples with higher porosity in the structure, this influence decreases. As explained in previous sections, even elevated temperature and pressure, when enough amount of the NaCl particles (in role of space holding agent) were used, cannot prevent NaCl agent particles cleaning. The influence of compression force on energy absorption is not significant. This indicated that pressure of 300 kN was enough in this case and increase to 500 kN do not have any additional influence. Of course, a wider range of compaction force, for example from 100 kN to 2000 kN, will probably yield some different results and conclusions. Predicted and actual values, shown in Figure 6b, are approaching each other. Expression (5) describes the quadratic equation for predicting the value of the response surface model for energy absorption at a strain of 50%, after insignificant terms are omitted.

(5) $$W\;at\;50\%\;deformation\;=\;112.72908-4.07989\cdot A+0.23927\cdot B+0.12525\cdot C-3.73446\cdot 10^{-3}\cdot A\cdot B+3.3248\cdot 10^{-2}\cdot A^{2}$$

### 3.4. Optimisation of Input Parameters

After the analysis of variance for all response values was performed, it was concluded that, in most analyses the samples with a lower volume percentage of NaCl in the structure proved to be optimal. In most analyses, compaction temperature was shown to be an influential factor that has mostly positive effects on the output variables as its value increases.

For a complete analysis and determination of the optimal solution, it is necessary to perform the optimization to determine the optimal sample with regard to previously defined conditions that must be met, so it is necessary to set the goal, lower and upper limit values for each input and the output variable. Table 7 shows the given conditions for finding the optimal pattern.

Numerical optimization balances input values for defined output variables. According to the problem being solved, the objectives are to maximize some responses, minimize others and collect ranges of input parameters according to the objectives that are defined. Assessment of maximum and minimum values is mandatory for responses. For the desired function value, weighting factors can be assigned. Furthermore, according to the needs and usefulness of the goals, the importance of some goals can be changed and considered. The desirability method finds the operating conditions that give the “most desirable” response values.

**Figure 7:**
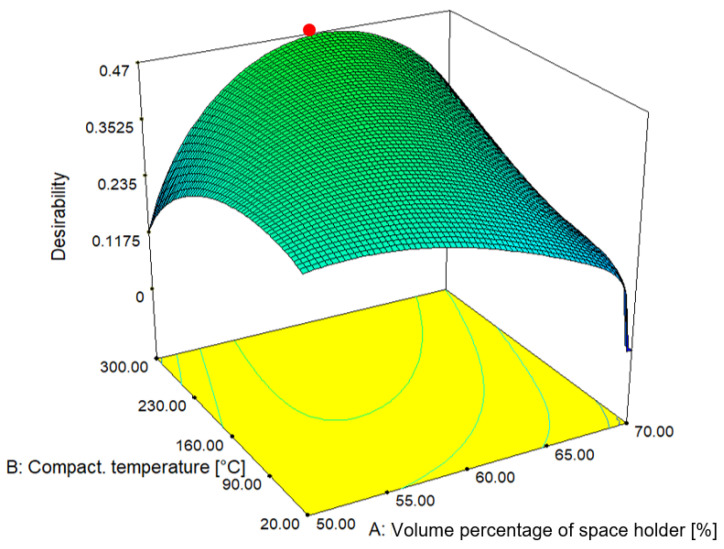
A 3D graph of desirability function with marked optimal parameters.

All output variables had the maximization of their values as the goal of optimization, except for the output variables of relative density, because its goal was to minimize them. In Figure 7, the 3D model of the surface is presented with regard to the set conditions and the obtained results. The area marked in green indicates the area of satisfactory results with regard to the set conditions and goals.

**Figure 8:**
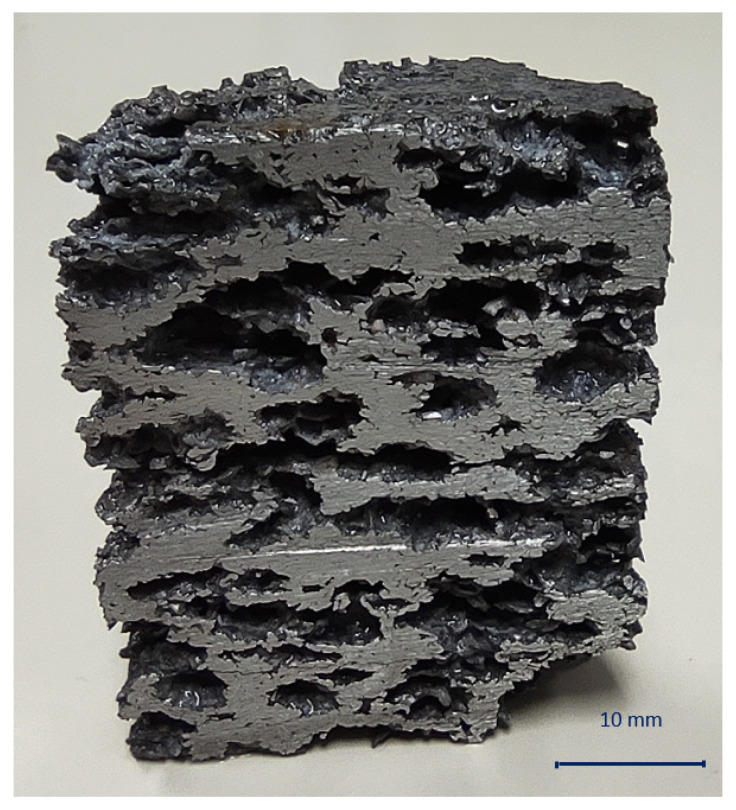
Cross section of the open-cell metal foams after space holder leaching.

As the optimal result, indicated by the red dot in Figure 6, a sample with 61.44% volume percentage of NaCl, a compaction temperature of 300 °C and a compaction force of 469 kN was determined by the numerical optimization. The metal foam produced in this way has a relative density of 0.511, stress and energy absorption at 50% deformation 72.53 MPa and 14.33 MJ/m^3^, respectively. The optimized results are above the “critically small” amount of volume percentage of space holder particles that indicated successful leaching of the agent was performed. If this has been achieved, there is no doubt that open-cell metal foams with interconnected cell voids have been obtained. If that was not a case, relative density will certainly be higher. Furthermore, as the optimization showed and as previously mentioned, elevated temperature and compaction force can be beneficial for achievement a stronger bond between aluminum chips and therefore higher-quality open-cell metal foams were produced with these parameters. Figure 8 shows a cross section of the foam on which space holder was dissolved.

### 3.5. Scanning Electron Microscopy with Energy Dispersive Spectroscopy Analysis

**Figure 9:**
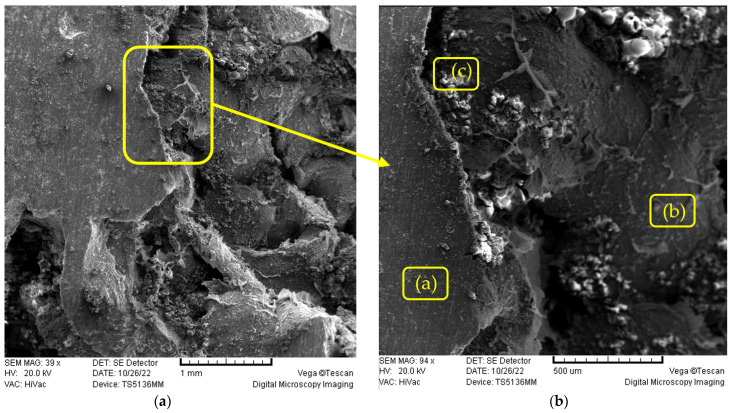
SEM images of metal foams. (**a**) microstructure of the samples produced by using optimal parameters, (**b**) enlarged part with three marked characteristic positions.

For metallography analysis, scanning electron microscope (SEM) and energy-dispersive X-ray spectroscopy (EDX) with technical designation TESCAN VEGA SEM device model 5136 MM were used. Figure 9a shows the microstructure of the sample produced with parameters obtained in the optimization process and Figure 9b shows enlarged part with three marked characteristic positions, which will be described in detail below.

**Figure 10:**
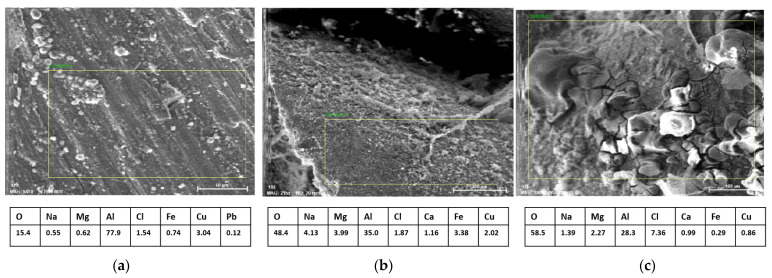
The energy dispersion spectroscopy (EDS) analysis of three different position marked in Figure 7: (**a**) metal foam cell wall; (**b**) metal foam pore; (**c**) corrosion on metal foam pore.

In Figure 10a, which presents the scanned surface for the EDS analysis of the metal foam, i.e., the cell wall, it is observed according to the micro-chemical composition that the space holder has been removed, and the presence of aluminum oxide is visible on the surface. All other chemical elements obtained by EDS analysis are approximately consistent with the basic chemical composition of the alloy given in Table 1. Deviations may result due to a randomly selected scanning position. Wan et al. [31] came to similar conclusions regarding the appearance of the oxide layer.

Figure 10b shows the appearance of metal foam pores. Considering the SEM image and the obtained micro-chemical composition, it can be concluded that the space holder was well washed out, but during removal process (temperature 100 °C, holding time 30 min) the conditions for initial strong corrosion were created. A low percentage of aluminum and a high percentage of oxygen suggests that aluminum oxides have formed. Increased Fe content may indicate the presence of iron oxide or an intermetallic compound (Fe-Al).

Figure 10c presents another interesting view into the pore itself. This image and EDS analysis show corrosion, as well as a cracked oxide layer, which is mainly composed of aluminum oxide and oxychloride. This suggested that the used method of metal foam production may have a negative impact on the corrosion properties of metal foam, and therefore an appropriate application should be selected following the mentioned conclusions.

## 4. Conclusions

In the present study, the feasibility of recycling aluminum alloy waste for the purpose of open-cell metal foam production was investigated. After experimental research, data processing and their comprehensive analysis, the following conclusions can be drawn:

(1) By analyzing the variance of the mathematical models for relative density, stress and energy absorption at 50% deformation, it was shown that the volume percentage of the pore-forming agent in metal foams is the most influential factor in the model, followed by compaction temperature. The force used to compact the sample is not significant for the mathematical model.
(2) By optimizing the processing parameters within the Design Expert 8 program, metal foam with a volume percentage of the space holder of 61.44%, compacted at a temperature of 300 °C with a compaction force of 469 kN proved to be the optimal solution. This type of foam has high stress and energy absorption values for reasonably low relative density.
(3) Finally, as a general conclusion, it can be stated that this process of metal foam production, which includes recycling waste in the form of chips obtained in the milling process of aluminum alloy workpiece, is a process that fully meets the principles of sustainable production. Unlike conventional aluminum alloy waste recycling procedures that re-melt the metal, the recycling in the presented research is carried out in the solid-state area, the maximum heating temperature is 300 °C and the agent used to create pores in metal foams is easily available and extremely low cost.
(4) The described process should be energetically, economically and environmentally more efficient than conventional aluminum recycling and metal foam production methods with significantly lower greenhouse gasses emissions.

## Figures and Tables

**Table 1:** Chemical composition of the aluminum alloy EN AW 2011 used for chips obtaining.

Si	Fe	Cu	Mn	Mg	Cr	Zn	Ti	Pb	Al
max. 0.4	max. 0.7	5.0–6.0	max 0.05	max 0.05	max. 0.05	max 0.3	max. 0.05	0.2–0.4	rest

**Table 2:** Input parameters.

Input Parameters	Minimal Value	Maximal Value
Volume percentage of space holder (%), A	50	70
Compaction temperature [°C], B	20	300
Compaction force [kN], C	300	500

**Table 3:** Experimental runs and responses.

	Input Parameters			Output Parameters		
Exp. Run	A (%)	B [°C]	C [kN]	*ρ_rel_*	*σ* at 50% Deformation [MPa]	*W* at 50% Deformation [MJ/m^3^]
1	63.00	20.00	500.00	0.43	19.03	4.41
2	70.00	300.00	300.00	0.34	16.57	3.61
3	70.00	133.40	418.97	0.36	11.73	2.49
4	62.59	195.00	500.00	0.47	53.80	8.84
5	70.00	300.00	300.00	0.38	18.11	3.84
6	58.05	300.00	419.70	0.59	93.54	19.90
7	70.00	300.00	500.00	0.37	12.94	3.12
8	70.00	20.00	300.00	0.33	6.38	1.34
9	62.50	20.00	371.96	0.42	27.99	4.88
10	50.00	20.00	371.07	0.57	42.25	14.4
11	50.00	300.00	300.00	0.66	161.70	34.16
12	50.00	200.12	500.00	0.65	111.85	28.90
13	57.61	125.00	424.50	0.48	28.16	6.75
14	58.08	132.92	300.00	0.50	33.15	7.96
15	70.00	20.00	300.00	0.33	7.58	1.50
16	68.10	273.99	400.00	0.40	21.97	4.16
17	70.00	300.00	500.00	0.37	28.07	2.86
18	50.00	300.00	300.00	0.71	89.16	29.50
19	70.00	133.40	418.97	0.36	13.18	2.67
20	50.00	20.00	500.00	0.59	28.39	11.53

**Table 4:** Analysis of variance for relative density.

Response 1 Relative Density						
ANOVA for Response Surface 2FI Model						
Source	Sum of Squares	df	Mean Square	F Value	*p*-Value	Prob > F
Model	0.28	6	0.046	153.85	<0.0001	significant
A	0.19	1	0.19	636.59	<0.0001	
B	0.015	1	0.015	51.07	<0.0001	
C	7.211 × 10^−4^	1	7.211 × 10^−4^	2.42	0.1438	
AB	4.668 × 10^−3^	1	4.668 × 10^−3^	15.67	0.0016	
AC	2.021 × 10^−5^	1	2.021 × 10^−5^	0.068	0.7986	
BC	1.783 × 10^−5^	1	1.783 × 10^−5^	0.060	0.8106	
Residual	3.873 × 10^−3^	13	2.980 × 10^−4^			
Lack of Fit	2.086 × 10^−3^	8	2.607 × 10^−4^	0.73	0.6714	
Pure Error	1.788 × 10^−3^	5	3.576 × 10^−4^			
Cor Total	0.28	19				

**Table 5:** Analysis of variance for stress at 50% deformation.

Response 2 Stress at 50% Deformation						
ANOVA for Response Surface 2FI Model						
Source	Sum of Squares	df	Mean Square	F Value	*p*-Value	Prob > F
Model	28,465.28	6	4744.21	16.28	<0.0001	significant
A	12,645.69	1	12,645.69	43.40	<0.0001	
B	8165.97	1	8165.97	28.03	0.0001	
C	166.07	1	166.07	0.57	0.4637	
AB	4199.48	1	4199.48	14.41	0.0022	
AC	65.57	1	65.57	0.23	0.6431	
BC	65.20	1	65.20	0.22	0.6440	
Residual	3787.67	13	291.36			
Lack of Fit	1039.23	8	129.90	0.24	0.9647	not significant
Pure Error	2748.44	5	549.69			
Cor Total	32,252.95	19				

**Table 6:** Analysis of variance for energy absorption at 50% deformation.

Response 3 Energy Absorption at 50% Deformation						
ANOVA for Response Surface Quadratic Model						
Source	Sum of Squares	df	Mean Square	F Value	*p*-Value	Prob > F
Model	1936.70	9	215.19	47.01	<0.0001	significant
A	1044.79	1	1044.79	228.24	<0.0001	
B	307.54	1	307.54	67.19	<0.0001	
C	8.32	1	8.32	1.82	0.2074	
AB	195.87	1	195.87	42.79	<0.0001	
AC	5.07	1	5.07	1.11	0.3172	
BC	0.12	1	0.12	0.026	0.8754	
A^2	32.91	1	32.91	7.19	0.0230	
B^2	5.44	1	5.44	1.19	0.3012	
C^2	2.32	1	2.32	0.51	0.4929	
Residual	45.78	10	4.58			
Lack of Fit	34.83	5	6.97	3.18	0.1149	not significant
Pure Error	10.95	5	2.19			
Cor Total	1982.47	19				

**Table 7:** The set conditions for finding the optimal input parameters.

Name	Goal	Lower Limit	Upper Limit	Lower Weight	Upper Weight	Importance
Volume perc. of space holder [%]	is in range	50	70	1	1	3
Compaction temperature [°C]	is in range	20	300	1	1	3
Compaction force [kN]	is in range	300	500	1	1	3
Relative density	minimize	0.329	0.707	1	1	5
Stress at 50% def. [MPa]	maximize	6.38	161.7	1	1	3
Energy absorb. at 50% def. [MJ/m^3^]	maximize	1.34	34.16	1	1	3

## Data Availability

Not applicable.
